# Development of a rapid, in‐situ analysis method using sheath‐flow probe electrospray ionisation‐mass spectrometry for the direct identification of cocaine metabolites in dried blood spots

**DOI:** 10.1002/rcm.9422

**Published:** 2022-11-16

**Authors:** Ayoung Kim, Paul F. Kelly, Matthew A. Turner, James C. Reynolds

**Affiliations:** ^1^ Centre for Analytical Science, Department of Chemistry Loughborough University UK

## Abstract

**Rationale:**

Small amounts of biofluid samples are frequently found at crime scenes; however, existing gold standard methods such as LC–MS frequently require destructive extraction of the sample before a time‐consuming analysis which puts strain on forensic analysis providers and can preclude further sample analysis. This study presents the application of sheath‐flow probe electrospray ionization‐mass spectrometry (sfPESI–MS) to the direct analysis of drug metabolites in dried blood spots (DBS) as a high throughput, minimally destructive alternative.

**Methods:**

A rapid direct analysis method using a sfPESI ionisation source coupled to an Orbitrap Exactive mass spectrometer was applied to detect cocaine metabolites (benzoylecgonine, BZE, cocaethylene, CE, and ecgonine methyl ester, EME) from DBS. An optimisation study exploring the use of different chemical modifiers (formic acid and sodium acetate) in the sfPESI probe extraction solvent was conducted to enhance the sensitivity and reproducibility of the sfPESI–MS method.

**Results:**

Optimisation of the extraction solvent significantly enhanced the sensitivity and reproducibility of the sfPESI–MS method. A quantitative response over a five‐point calibration range 0.5 to 10 μg/ml was obtained for BZE (R^2^ = 0.9979) and CE (R^2^ = 0.9948). Limits of detection (LOD) of 1.31, 0.29 and 0.15 μg/ml were achieved for EME, BZE and CE, respectively, from 48 h aged DBSs with % RSD (relative standard deviation) across the calibration range ranging between 19%–28% for [BZE + H]^+^, 13%–21% for [CE + H]^+^ and 12%–29% for [EME + H]^+^.

**Conclusions:**

A rapid (< 20 s) quantitative method for the direct analysis of cocaine metabolites from DBS which requires no prior sample preparation was developed. Although the LOD achieved for BZE (LOD: 0.29 μg/ml) was above the UK threshold limit of exposure for drug driving (0.05 μg/ml), the method may be suitable for use in identifying overdose in forensic analysis.

## 1 INTRODUCTION

Biological samples such as dried blood spots (DBSs) can provide useful information in both criminal investigations and identifying accidental or deliberate exposure to chemicals. The presence of drugs and/or their metabolites in a sample can give additional information about a suspect, perpetrator or victim in a criminal investigation or be used to identify the chemical agent responsible for intoxication or poisoning. In a forensic setting though several different bodily fluids are commonly found in crime scene investigations, blood is the most widely studied forensic biofluid; however, other fluids such as saliva, semen, urine, sweat and vaginal fluid have also played a vital role in specific investigations.^1^ The identification of a biofluid at a crime scene usually will begin with a presumptive test, which is typically done on site to determine the identity of the stain, before sending the sample to a specialist laboratory for confirmatory analysis, including DNA profiling, to potentially identify the suspect.^1^, ^2^ Presumptive tests are typically simple colorimetric tests that indicate the presence of a particular fluid by a colour change which is induced by the addition of a reagent (e.g. luminol solution reacting with iron in blood),^3^ with each body fluid requiring a specific presumptive test to enable identification.^1^ There are limitations which apply to presumptive tests; some may lack specificity and react with multiple biofluids or other chemical interferents which may be present at the scene causing false‐positive results.^4^ In addition, many common presumptive tests are destructive to the sample and may render it useless for subsequent confirmatory tests and/or DNA analysis.^1^ This is a particular issue when small volumes of the sample are present.^5^

In recent years, analytical methods have been developed to address this issue, and a range of different spectroscopic methods have been applied to identify body fluids. These methods include Raman spectroscopy^6^, ^7^, ^8^ and Fourier transform‐infrared spectroscopy (FT‐IR)^8^, ^9^ both of which are non‐destructive methods that will not damage the sample in the way a conventional presumptive test may. Mass spectrometry (MS) is a particularly attractive technique for biofluid identification and screening as it possesses a high degree of sensitivity and low limits of detection, and can identify low‐abundance analytes in a biofluid sample.^10^, ^11^ This gives a mass spectrometric method the ability to gain additional information about the biofluid sample and the individual it originated from, in addition to identifying what the biofluid is. For example, the presence of pharmaceuticals, narcotics or poisons in the biofluid sample can be determined, which may be beneficial to the investigation in giving further insight into the crime being committed and reduce the load on testing laboratories.^12^ In the confirmatory testing role, MS has long been a gold‐standard method for analysing illicit substances in body fluids. However, the majority of standard methods and well‐used approaches such as liquid chromatography‐mass spectrometry (LC–MS) require destructive extraction, modification and preconcentration techniques to be employed before analysis.^1^, ^13^

Ambient ionization MS methods are capable of analysing samples in‐situ with minimal sample preparation and have been used both to identify biofluids^14^, ^15^ and to detect specific targeted compounds present in biofluids, including drug compounds^16^, ^17^ and disease biomarkers.^17^, ^18^ Probe electrospray ionization (PESI), a method developed by Hiraoka et al at the University of Yamanishi in Japan,^19^ is a particularly valuable method for forensic biofluid analysis as it requires no sample preparation and has short analysis times. In addition, PESI has been shown to consume only a few picolitres of sample during analysis which makes it useful in forensic scenarios where only trace levels of sample may be present.^20^ PESI has been applied to analyse a range of analytes, including the detection of illicit drugs in body fluids.^21^ In such cases the PESI method was shown to be able to detect cocaine with a limit of detection (LOD) of 0.05 μg/ml in 1:1 diluted urine, 0.02 μg/ml in 1:1 diluted oral fluid and 0.2 μg/ml in plasma after a protein precipitation step, with good quantitative performance (% RSDs [relative standard deviation] of 9.55% in urine, 10.44% in oral fluid and 14.81% in plasma).^21^ A limitation of the PESI method is that it is not capable of analysing solid samples, which restricts its utility towards samples such as DBS. A subsequent development of the PESI technique, sheath‐flow PESI (sfPESI) enabled the direct analysis of solid samples.^22^, ^23^ A sfPESI interface uses a fine needle contained within a solvent‐filled gel‐loading tip, with the needle protruding slightly (*c*. 0.1 mm) from the base of the gel‐loading tip.^23^, ^24^, ^25^ Analytes are then extracted from a surface by touching the sfPESI probe to the sample surface, which can be either a wet or dried material, for 5 s in which liquid extraction from the surface occurs.^25^ A high voltage (≤ 2.5 kV) is applied to facilitate an electrospray through the probe for a short duration, *c*. 5 s.^22^, ^23^, ^24^, ^25^, ^26^ There are a number of advantages in this method. Due to the small diameter of the needle of the sfPESI probe, only about 1 mm^2^ of the sample surface (Figure S1b [supporting information]) is affected by the extraction making it minimally destructive. Most notably probe electrospray methods, including sfPESI, exhibit a sequential ionization mechanism where analytes and matrix compounds show a degree of separation based on their surface activity.^23^, ^27^ This study showed that when a cytochrome C protein sample was mixed with a 100‐fold excess of a non‐ionic detergent (Triton X100) and analysed using PESI.^27^ The highly surface active detergent was emitted in the first 10 s of the analysis, whereas the less surface active protein was emitted later between 30 and 50 s, enabling improved detection of the protein as it reduced ion suppression by the Triton X100.^27^ When the same sample was analysed using nano‐electrospray, severe ion suppression of the cytochrome C caused by the presence of the detergent was noted.^27^ The same study also used PESI to analyse the peptide hormone insulin in the presence of an excess of NaCl. In this example, the peptide was eluted first with the Na^+^ ions only being electrosprayed at the final stage of the electrospray implying enrichment of NaCl in the main droplet on the PESI emitter over time. NaCl clusters were observed only in the final stages of the analysis which showed an almost‐complete separation of the peptide from the salt. These data confirm that the ionization of solvated analytes in PESI occurs in the order of decreasing surface activity.^27^ sfPESI‐MS has previously been used to identify biofluid samples using a profiling approach which was able to distinguish blood, urine, saliva and semen in both fresh and dried samples, demonstrating the ability of sfPESI to extract small metabolites like creatinine, carnitine and urea as well as larger phospholipids.^25^, ^28^ That study also investigated sample ageing, and it was noted that responses associated with certain phospholipid ions decreased as the DBS aged.^25^ Although demonstrating an ability to profile biofluids, however, this method has not yet been applied to perform quantitative measurements of compounds such as drug metabolites in dried samples.

In this study, sfPESI‐MS is applied to analyse cocaine metabolites (benzoylecgonine, BZE, cocaethylene, CE, and ecgonine methyl ester, EME) directly from 48 h aged DBS. A continuous flow sfPESI probe configuration is demonstrated, enabling high sample throughput, and the addition of chemical modifiers (an acidic and alkali metal modifier) into the sfPESI probe extraction solvent is investigated to facilitate quantitative analysis of cocaine metabolites directly from a DBS sample matrix.

## 2 EXPERIMENTAL

### 2.1 Materials

Cocaine metabolite standards, BZE (1.0 mg/ml in methanol), CE (1.0 mg/ml in acetonitrile) and EME (1.0 mg/ml in acetonitrile) were purchased from Sigma‐Aldrich (Cerilliant, Gillingham, UK). A mixture of water (HPLC grade, VWR Chemicals, France) and ethanol (99.98% absolute, VWR Chemicals, France) was used as an extraction solvent for the sfPESI source. Methanol (99.8% HPLC grade, Fisher Chemical, Loughborough, UK) was used to dilute standard samples for sfPESI probe optimisation tests. Formic acid (FA, 95% Liquid, Sigma‐Aldrich, Germany) and sodium acetate (SA, 99% Powder, Sigma Aldrich, Germany) were used as chemical modifiers for the extraction solvent. Whole human blood samples were taken by venous draw, from a single consenting volunteer using a protocol approved by the ethical committee of Loughborough University (Ethics statement number R18‐P034). The blood samples were aliquoted out and then spiked with each cocaine metabolite standard into a consistent amount of whole blood before spotting on to glass microscope slides (25.4 × 76.2 × 1.2 mm, Sail Brand, China). The blood spot specimens were stored in a dark cupboard at room temperature (22 ± 1°C) for 48 h to enable them to dry prior to sfPESI‐MS analysis.

### 2.2 sfPESI emitter construction and interfacing

**FIGURE 1:**
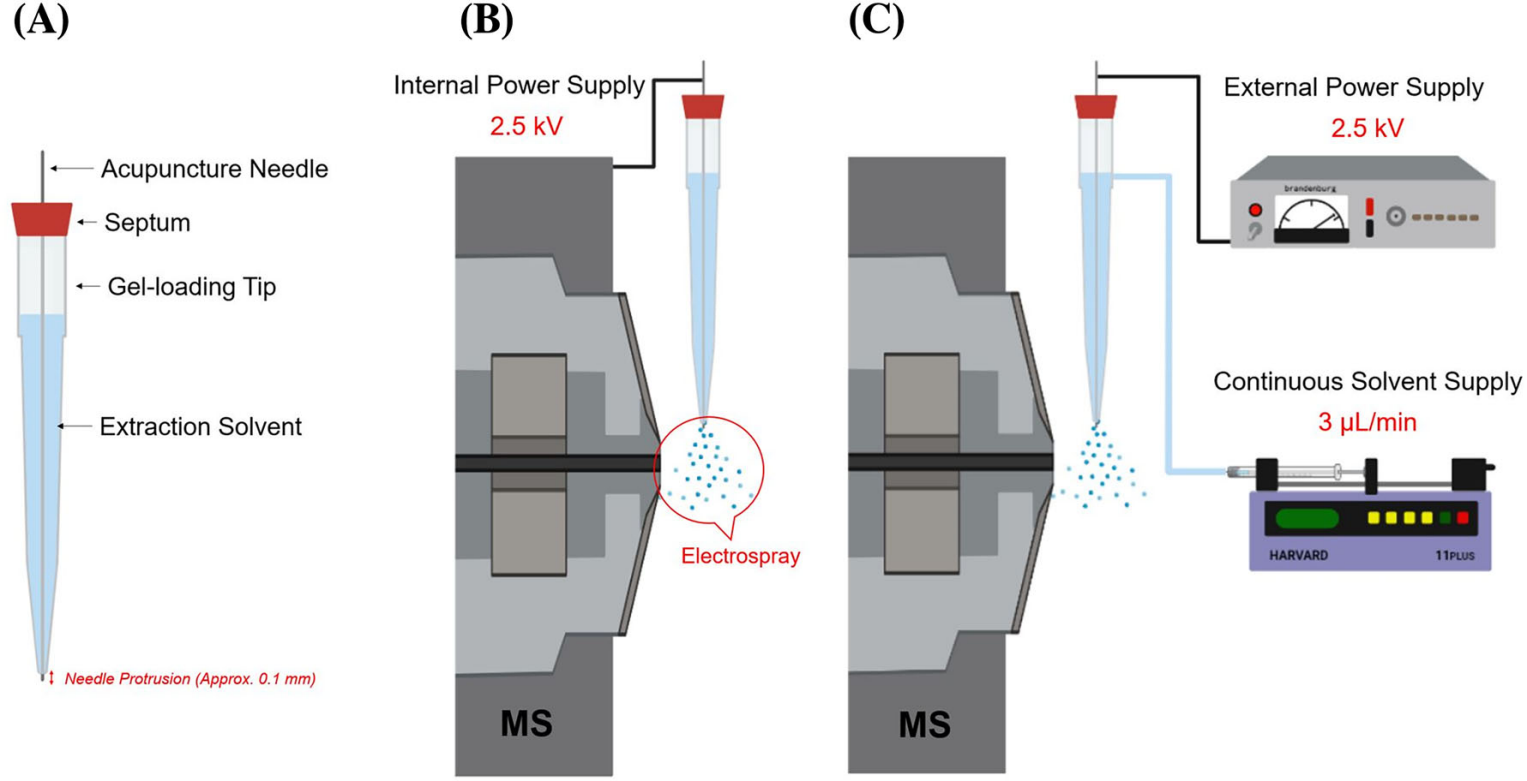
Sheath‐flow probe electrospray ionisation (sfPESI) emitter design and source configuration (created with biorender.com): A, structure of the sfPESI emitter, B, initial sfPESI configuration and C, modified continuous flow sfPESI interface [Color figure can be viewed at wileyonlinelibrary.com]

The sfPESI source (Figure 1) was constructed from a gel‐loading pipette tip (0.5–20 μl GELoader tip, Eppendorf AG, Hamburg, Germany) and a fine stainless steel acupuncture needle (J type OD 0.12 mm × L 30 mm, Seirin, Shizuoka, Japan) which was inserted into the gel‐loading tip (Figure 1A). The gel‐loading tip was filled with *c*. 30 μl extraction solvent. The acupuncture needle was held by a silicone septum (11 mm Non‐Stick BTO septa, Restek, Saunderton, UK) at the top of the gel‐loading tip and protruded *c*. 0.1 mm from the end of the gel‐loading tip. The acupuncture needle acted as an electrical conductor to generate an electrospray from the mobile phase when a high voltage (2.5 kV) was applied.

Initial sfPESI‐MS data for the extraction solvent efficiency study were acquired using the interface configuration shown in Figure 1B, which has been reported elsewhere.^25^ Although this configuration can obtain good quality sfPESI‐MS data, it introduces variability in replicate measurements due to the position of the emitter with respect to the MS inlet varying when the emitter is manually refilled, which also reduces sample throughput. To address these issues an sfPESI emitter was interfaced with an external power supply unit (292R High Voltage Power Supply, Brandenburg [now Genvolt], Bridgnorth, UK) and a continuous 3 μl/min solvent supply using a syringe pump (11 Plus, Harvard Apparatus, Hollistion, MA, USA) and a 500 ml syringe (SGE 500R‐GT‐LC [SS], Sigma‐Aldrich, Gillingham, UK) (Figure 1C). The continuous solvent supply reduced variability and provided consistency by fixing the position of sfPESI probe enabling multiple samples to be compared without adjusting the sfPESI probe position between measurements. It also reduced the time‐consuming work of having to manually dismantle, refill and rebuild the emitter between samples when performing replicate sfPESI‐MS analyses.

### 2.3 sfPESI‐MS analysis method

Initial sfPESI‐MS experiments were performed using a 50%v/v ethanol/water solution as the extraction solvent; however, after an optimisation study, a 50%v/v ethanol/water solution containing 0.5mM sodium acetate (SA) and 0.1% formic acid (FA) was used as an optimised extraction solvent for the sfPESI emitter.

For the optimisation study, liquid sample solutions of 5 μg/ml BZE were analysed by dipping the emitter into the solution. For DBS analysis, samples were extracted from DBS by gently touching the sfPESI emitter onto the sample surface for 5 s, which allowed the extraction of analytes from the surface to occur by capillary phenomena, with an area of < 1 mm^2^ being sampled (Figure S1 [supporting information]). In addition, for quantitative experiments, a background blank signal was measured by extracting directly from the glass slide surface before commencing DBS analysis, and additional background blank samples were analysed between DBS analyses to ensure no carryover occurred between each replicate DBS analysis.

A Thermo Exactive Orbitrap mass spectrometer (ThermoFisher Scientific, Bremen, Germany) was used for all sfPESI‐MS analyses, and the optimised settings of the mass spectrometer are shown in Table S1 (supporting information). The sfPESI emitter was vertically fitted in front of the Orbitrap mass spectrometer with the emitter positioned with the distance of *c*. 3 mm from the MS inlet. A high voltage of 2.5 kV by an external power supply (292R High Voltage Power Supply, Brandenburg (Genvolt), Bridgnorth, UK) was then applied for 5 s to facilitate an electrospray. The scan rate was 2.4 scans per second.

### 2.4 Data analysis

Data were collected and analysed using Thermo Xcalibur software (Version 3.0, ThermoFisher Scientific, Bremen, Germany) with a < 5 ppm mass error (or mass tolerance). Mass spectral intensities were obtained after subtraction of the background mass spectrum from the glass slide from the DBS mass spectrum to remove background ions which originate from either the glass slide surface or the extraction solvent. From the mass spectral data acquired, fundamental statistics, such as the mean and relative standard deviation (% RSD) of mass spectral intensities obtained, were used to compare the results.^29^ Outliers were rejected using Dixon's Q‐test when assessing the reliability of analysis results, and a minimum of six replicates were measured for each analytical sample to reduce random error and improve the precision of data obtained.^29^ Limits of detection (LOD = 3*σ/m*) were calculated by taking thrice the calculated standard deviation (*σ*) in the sample response at the lowest sample concentration and dividing by the slope value (*m*) of the linear correlation graphs generated.^29^ All graphs were created and analysed through the Origin software (Version 2019, OriginLab Corporation, USA).

## 3 RESULTS AND DISCUSSION

sfPESI‐MS was applied to analyse cocaine metabolites (BZE, CE and EME) directly from DBSs as a rapid, direct and minimally destructive analysis method with no requirement for prior sample preparation. A list of the cocaine metabolites under investigation with their molecular structures and expected adduction forms in sfPESI‐MS analysis are shown in Table 1. Prior to commencing analysis of cocaine metabolites from DBS, an optimisation study was performed using liquid standard samples to improve the sensitivity and reproducibility of the sfPESI‐MS method.

**TABLE 1:** Molecular structures and masses of cocaine metabolites

Cocaine metabolite	Molecular structure	Mass^a^		Adducted ion form
Benzoylecgonine (BZE)	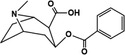	RMM 289	*m/z* 290	[M + H]^+^ ^b^
			*m/z* 312	[M + Na]^+^ ^c^
			*m/z* 328	[M + K]^+^ ^d^
Ecgonine methyl ester (EME)	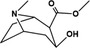	RMM 199	*m/z* 200	[M + H]^+^
			*m/z* 222	[M + Na]^+^
			*m/z* 238	[M + K]^+^
Cocaethylene (CE) or Ethylbenzoylecgonine (EBZE)	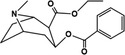	RMM 317	*m/z* 318	[M + H]^+^
			*m/z* 340	[M + Na]^+^
			*m/z* 356	[M + K]^+^

^a^ Mass: the mass calculated from a molecular formula using the most abundant isotopes and expressed by mass‐to‐charge ratio (m/z).

^b^ [M + H]^+^: singly charged protonated molecular positive ion.

^c^ [M + Na]^+^: singly charged sodiated molecular positive ion.

^d^ [M + K]^+^: singly charged potassiated molecular positive ion.

### 3.1 Extraction solvent chemical modifier optimisation

An aqueous 50%v/v ethanol/water solution was initially used as an extraction solvent. About 5 μg/ml of a BZE standard solution in methanol was analysed as a liquid sample to avoid any variability caused by sample inhomogeneity from drying onto a surface. Ten replicate analyses were carried out to evaluate the sensitivity and precision (% RSD) of the sfPESI‐MS method with each different set of conditions. The sfPESI method shows an almost‐instantaneous response for the cocaine metabolites, which are detected within 1 s of initiating the spray voltage and exhausted in 3 s. Other species including background interferences and cluster ions are emitted and detected for *c*. 5 s, demonstrating the speed and potential high throughput of the method.

Three intense peaks which were attributed to BZE were detected in the mass spectra obtained under these conditions (Table 2). The base peak was a sodiated BZE ion [M + Na]^+^ at *m/z* 312 with a potassiated BZE ion [M + K]^+^ detected at *m/z* 328 and a protonated BZE ion [M + H]^+^ observed at *m/z* 290. The alkali metal adduct ions were detected in this test even though no chemical modifier for the extraction solvent was added. In positive ionization mode of electrospray ionization‐mass spectrometry (ESI‐MS), the most common alkali metals readily producing adduct ions are sodium (Na) and potassium (K) due to trace‐level alkali metals being present in solvents and glassware used for preparing samples according to prior research.^30^ However, controlling these adducted species without chemical modifiers is not possible because the alkali metals and their compounds are irregularly present in the mobile phase and sample solution. Based on these initial results (Table 2) it is evident that although sfPESI‐MS was capable of detecting BZE ions reliably from a liquid standard solution, the response is highly variable with % RSD of 79% for the [M + H]^+^ ion, 72% for the [M + Na]^+^ ion and 80% for the [M + K]^+^ ion. Therefore, chemical modifiers, such as FA to promote formation of [M + H]^+^ ions and SA to promote formation of [M + Na]^+^ ions, were investigated to enhance the mass spectral response and improve the precision of the measurement.

**TABLE 2:** Ion responses and percentage relative standard deviation (n = 10) for benzoylecgonine (BZE) liquid standards in methanol using 50:50%v/v ethanol:Water extraction solvent

Molecular ion form		[M + H]^+^	[M + Na]^+^	[M + K]^+^
Mass		*m/z* 290	*m/z* 312	*m/z* 328
Intensity (c/s)	M^a^ (n = 10^b^)	1.42.E+05	4.33.E+05	7.79.E+04
	STDEV^c^	1.12.E+05	3.11.E+05	6.23.E+04
	% RSD^d^	79	72	80

^a^ M: mean or average.

^b^ n: the number of measurements.

^c^ STDEV: standard deviation.

^d^ % RSD: percentage relative standard deviation.

Adding an acidic modifier (0.1% and 0.5% FA, Table S2 [supporting information]) showed a significant increase in mass spectral intensity of the [M + H]^+^ with and an approximately 30‐fold increase in intensity when 0.1% FA was added to the extraction solvent. However, there was a commensurate decrease in the intensity of the alkali metal adduct ions, [M + Na]^+^ and [M + K]^+^. Adding 0.5% FA into the extraction solvent showed a lower overall intensity compared to 0.1% FA. The repeatability of the method was improved when FA was added, like % RSD precision improving to 43% when 0.1% FA was used, with 0.5% FA performing worse with % RSD of 58%. Although the improvement in signal intensity by adding an acid modifier showed significantly enhanced sensitivity, the method still produced relatively variable results and thus the effect of alkali metal modifiers was explored, both with and without adding a positive charge modifier to obtain higher analytical precision.

To explore the effect of applying an alkali metal modifier, SA was considered which has in previous studies been shown to stabilise drug metabolites through the formation of stable sodium adducts.^31^ The addition of 0.5mM of SA into the extraction solvent resulted in a substantial enhancement in average mass spectral intensity for the [M + Na]^+^ ion of BZE which was observed as the base peak in the spectrum while the [M + H]^+^ and [M + K]^+^ ions were significantly suppressed. The reproducibility of the mass spectral intensities of the [M + Na]^+^, [M + H]^+^ and [M + K]^+^ ions improved with % RSDs of 32%, 24% and 38%, respectively (Table S3 [supporting information]).

The use of chemical modifiers in the sfPESI probe extraction solvent will influence its properties when extracting multifarious species from various sample surfaces, and the extraction efficiency will depend on analyte characteristics and molecular structure. Applying a combination of acidic and basic alkali metal modifiers to the extraction solvent was investigated. A mixture of chemical modifiers (0.1% FA and 0.5mM SA) in 50% ethanol aqueous solution was, therefore, considered. Using this solution as the extraction solvent compared to 50%v/v ethanol/water solution, an improvement was achieved in both the mass spectral intensities and % RSD precision for all three cocaine metabolites (Table S4 [supporting information]), [BZE + H]^+^ 3.3‐fold increase in intensity with % RSD of 32% (no modifiers 81%), [BZE + Na]^+^ 3.3‐fold increase in intensity with % RSD of 39% (no modifiers 65%), [EME + H]^+^ 2.3‐fold increase in intensity with % RSD of 30% (no modifiers 55%), [CE + H]^+^ 2.2‐fold increase in intensity with % RSD of 36% (no modifiers 99%). Due to the improved reproducibility of the results obtained using the combination of acidic and alkali metal modifiers compared to using an acidic modifier alone, this extraction solvent was selected to be used for quantitative DBS analysis.

### 3.2 Application to DBS analysis

Human whole blood generally provides a complex background including many different species, such as proteins, lipids, salts, metabolites, during ESI‐MS analysis in either positive or negative ion mode, making the native blood sample a challenging matrix, and frequently requiring extensive sample preparation to enable measurements of targeted metabolite species.^25^ For direct analysis of DBS containing cocaine metabolites, the extraction solvent containing both modifiers, 0.1% FA and 0.5mM SA, was compared to the 50%v/v ethanol/water extraction solvent to determine whether reproducible data could be acquired from DBS.

Six sets of 48 h DBS spiked with 10 μg/ml of each of the three cocaine metabolites (BZE, CE and EME) were used as test specimens. The blood spot samples were stored in a dark cupboard at room temperature (22°C ± 1) until sfPESI‐MS analysis was performed. Metabolites were extracted from the surface using either 50%v/v ethanol/water or 50%v/v ethanol/water + 0.1% FA and 0.5mM SA solution as the sfPESI probe extraction solvent. The average noise level of the mass spectra was *c*. 7000 c/s by absolute abundance. The data acquired while showing significantly lower ion responses than that observed from analysing liquid solutions demonstrated that all three metabolites could be successfully extracted from the surface and detected. When comparing the two extraction solvents, the increase in ion response was much less marked when analytes extracted from DBS were considered rather than liquid solutions. However, there was a significant improvement in the reliability and stability of mass spectral responses when using the modified extraction solution (Table 3). When 0.1% FA and 0.5mM SA were added into the extraction solvent for the sfPESI probe, % RSD precision for all masses monitored was improved with % RSDs of 42% (no modifiers 70%) for [BZE + H]^+^, 26% (no modifiers 30%) for [BZE + Na]^+^, 14% (no modifiers 70%) for [EME + H]^+^ and 28% (no modifiers 45%) for [CE + H]^+^ (Table 3). The improvement in reproducibility with the use of the mobile phase modifier may originate from improved control of the pH during the sample extraction and analysis giving more reliable protonation of the analytes. In addition, the localisation of excess charges in the Taylor cone and the resulting droplets formed during the electrospray process may also play a role as described by Hiraoka et al^32^ The metabolite ions are the most surface active compounds present in the liquid sample and will localise to the surface of the liquid and be emitted first in the sfPESI analysis. Hydronium ions (H_3_O^+^) have a high hydration free energy (93.3 kcal/mol) compared to Na^+^ (87.3 kcal/mol) and K^+^ (70.5 kcal/mol) ions. This means Na^+^ and K^+^ are more surface active than H_3_O^+^ by 6 kcal/mol and 16.8 kcal/mol, respectively, and therefore Na^+^ and K^+^ will localise to the surface of the Taylor cone during the electrospray process, and H_3_O^+^ ions as the least surface active species will remain in the bulk liquid for long time.^32^ This effect will lead to the formation of Na^+^‐enriched droplets during the electrospray process.^32^ Na^+^ and K^+^ ions will be extracted from the DBS by the sfPESI probe extraction solvent and will be a source of variability between replicate analyses. The addition of excess Na^+^ ions into the extraction solvent through the addition of SA minimises the contribution from Na^+^ and K^+^ ions present in the DBS, thus reducing the variability observed in the replicate analyses when modifiers were used.^32^ These data show the benefit of using chemical modifiers in mobile phase when performing direct analysis from DBSs using sfPESI and demonstrate the potential for quantitative analysis.

**TABLE 3:** Comparative data from three cocaine metabolites directly analysed from dried blood spots using 50%v/v ethanol:water and 50%v/v ethanol:water + 0.1% formic acid (FA) and 0.5mM sodium acetate (SA)

Molecular ion form Target mass Chemical modifier		[BZE + H]^+^ ^a^		[BZE + Na]^+^ ^b^		[EME + H]^+^ ^c^		[CE + H]^+^ ^d^	
		*m/z* 290		*m/z* 312		*m/z* 200		*m/z* 318	
		None	0.1% FA 0.5mM SA	None	0.1% FA 0.5mM SA	None	0.1% FA 0.5mM SA	None	0.1% FA 0.5mM SA
Intensity (c/s)	M (n = 6)	6.32.E+04	1.46.E+05	5.10.E+04	8.47.E+04	1.90.E+05	1.13.E+05	7.08.E+05	7.16.E+05
	STDEV	4.42.E+04	6.14.E+04	1.52.E+04	2.18.E+04	1.34.E+05	1.56.E+04	3.17.E+05	2.03.E+05
	% RSD	70	42	30	26	70	14	45	28

^a^ [BZE + H]^+^: singly charged protonated benzoylecgonine ion.

^b^ [BZE + Na]^+^: singly charged sodiated benzoylecgonine ion.

^c^ [EME + H]^+^: singly charged protonated ecgonine methyl ester ion.

^d^ [CE + H]^+^: singly charged protonated cocaethylene ion.

### 3.3 Surface activity separation

A common issue that can arise when performing direct analysis on complex biofluids using ESI, and particularly when using mixed modifiers, is that the presence of background ions and undesired adducts can act as matrix interferences.^33^, ^34^ These interferences can mask analyte signals through the formation of matrix ion clusters^33^ and reduce the overall abundance of analytes through ion suppression.^34^ Using the solvent modifiers (FA and SA) shown earlier, sodium formate clusters will be readily formed through electrospray ion formation process when sodium formate is formed by nebulizing formate with sodium.^35^ Sodium formate cluster ions, [NaOOCH]_n_ Na^+^ (1 ≤ n), can be produced from the carboxylate anions [HCOO]^−^ and sodium cations [Na]^+^.^35^ The [NaOOCH]_n_ Na^+^ (1 ≤ n) ions with bonding order (n) between 1 and 7 were monitored as they covered the scan range from *m/z* 50 to 450 that the cocaine metabolites were analysed over in this study. It is possible to detect over this range 7 of these cluster ions which were observed at *m/z* 90.98 (n = 1), *m/z* 158.96 (n = 2), *m/z* 226.95 (n = 3), *m/z* 294.94 (n = 4), *m/z* 362.93 (n = 5) and *m/z* 430.91 (n = 6) and *m/z* 498.90 (n = 7), respectively.

**FIGURE 2:**
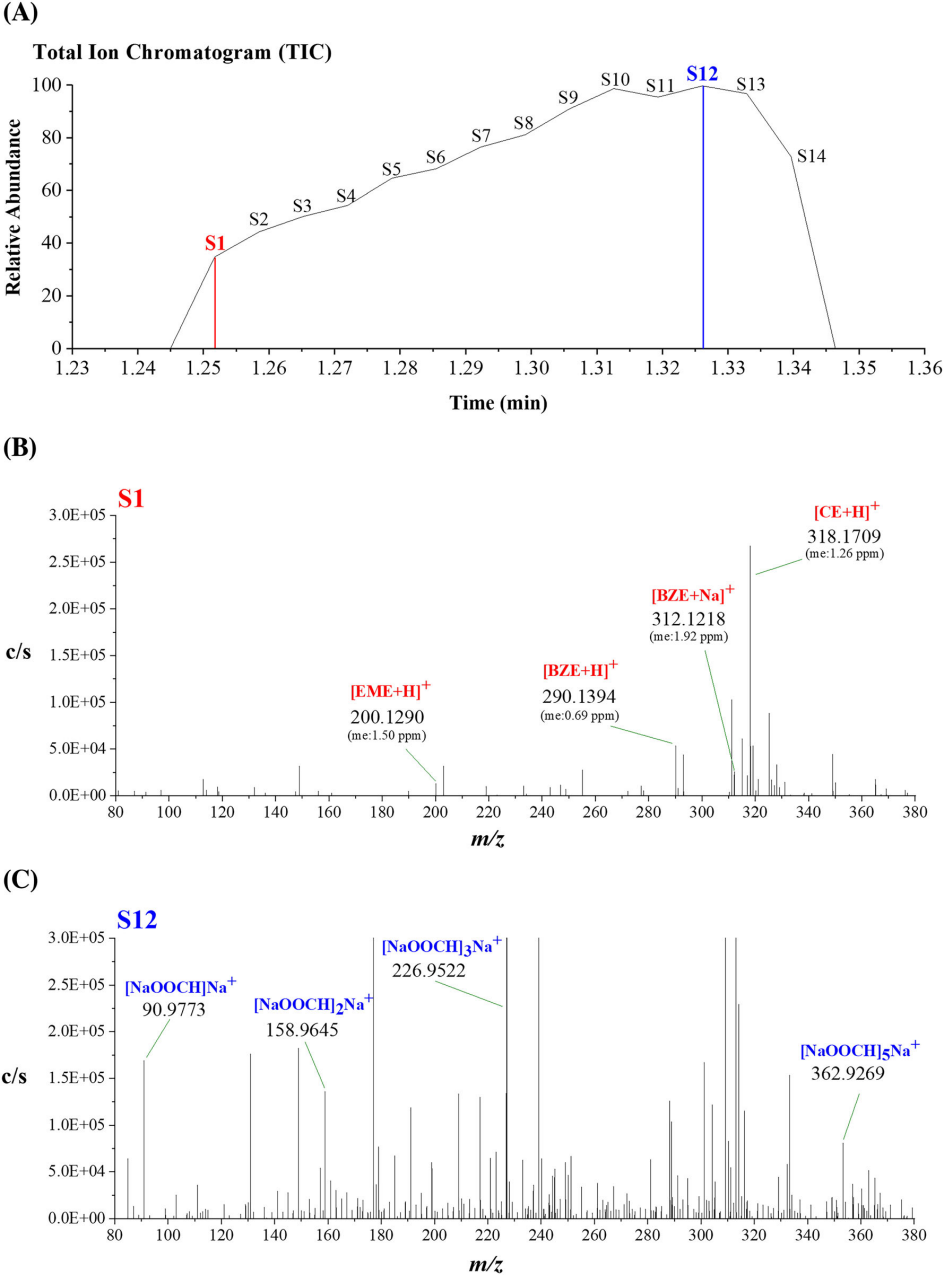
Analysis result of dried blood spots spiked by 10 μg/ml of cocaine metabolites (benzoylecgonine, BZE, cocaethylene, CE, and ecgonine methyl ester, EME): A, 14 scans of analyte total ion chromatogram (TIC), B, mass spectrum of the 1st scan (S1) of TIC for cocaine metabolites (ms: mass error or mass tolerance ≤ 5 ppm) and C, mass spectrum of the 12th scan (S12) of TIC for sodium formate clusters [Color figure can be viewed at wileyonlinelibrary.com]

One cycle of measurement including extraction and sfPESI‐MS of both the background and the DBS took *c*. 20 s in total. The data from the DBS sample were acquired over 6 s (Figure 2); in this example, a total of 14 scans were obtained from the total ion chromatogram (TIC) of DBS using a scan rate of 2.4 scans/s. The [M + H]^+^ ions of the cocaine metabolites EME, BZE and CE were detected at *m/z* 200.1290 (1.5 ppm mass error), 290.1394 (0.7 ppm mass error) and 318.1709 (1.3 ppm mass error), respectively. The [M + Na]^+^ ion of BZE was detected at *m/z* 312.1218 (1.9 ppm mass error). In addition to the cocaine metabolites several sodium formate cluster ions, [NaOOCH]_n_Na^+^ (1 ≤ n ≤ 7), at *m/z* 90.9773, 158.9645, 226.9522 and 362.9269 were detected. Figure 2 shows that in the mass spectrum obtained from the first scan (S1), which appears < 1 s after applying the spray voltage of 2.5 kV, the cocaine metabolite ions are the dominant species without sodium formate cluster interferences being observed and with minimal interferences from background ions (Figure 2B). However, at the second time point (the 12th scan, S12 at about 5 s) the spectrum is dominated by background ions and the sodium formate clusters are clearly observed (Figure 2C).

**FIGURE 3:**
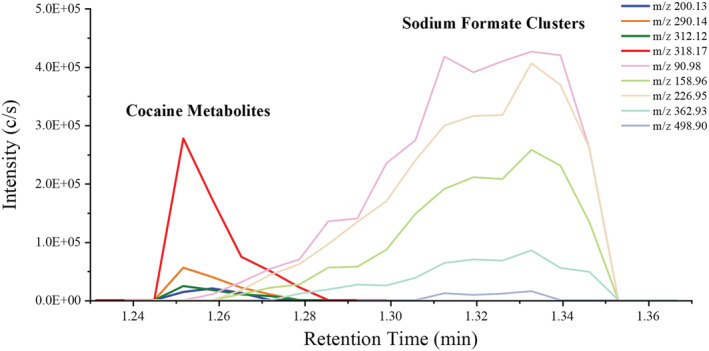
Comparison result of extracted ion chromatograms (EIC) between cocaine metabolites and sodium formate clusters, [NaOOCH]n Na^+^ (1 ≤ n ≤ 7): the cocaine metabolite ions (*m/z* 200.13, 290.14, 312.12 and 318.17) and the [NaOOCH]n Na^+^ ions (*m/z* 90.98, 158.96, 226.95, 362.93 and 498.90) [Color figure can be viewed at wileyonlinelibrary.com]

This rapid separation mechanism is a known advantageous feature of PESI methods, and it was firstly described by Mandal et al in 2011 who related the sequential and exhaustive ionization of a range of different analytes and contaminants to the different surface activity values of each analyte in PESI analysis.^27^ This sequential ionization method enabled the analysis of samples containing biological material and contaminants, like surfactants and various salts, without any prior clean‐up stage as the different surface activity values of all ionizable components provided different time points at which the analytes were emitted from the PESI source, thus providing a rapid clean‐up of the mass spectra.^27^ When comparing the extracted ion chromatograms (EICs) of the adducted cocaine metabolite ions (*m/z* 200.1290, 290.1394, 312.1218 and 318.1709) with the [NaOOCH]_n_ Na^+^ ions (*m/z* 90.9773, 158.9645, 226.9522 and 362.9269), the sequential ionization mechanism of sfPESI can be clearly demonstrated as shown in Figure 3. Figure 3 shows that the sodium formate clusters can be partially resolved from the cocaine metabolites within the 6 s analysis time, achieving a rapid separation where the clusters do not significantly interfere with cocaine metabolites, improving spectral interpretation and quantitative performance. The elution order achieved here agrees with the earlier work by Mandal *et al* (2011) showing that the more surface active compounds (e.g., cocaine metabolites) elute at the beginning of the electrospray event, whereas the Na^+^ ions remain in the solvent longer and are emitted in the final stages of the electrospray.

**FIGURE 4:**
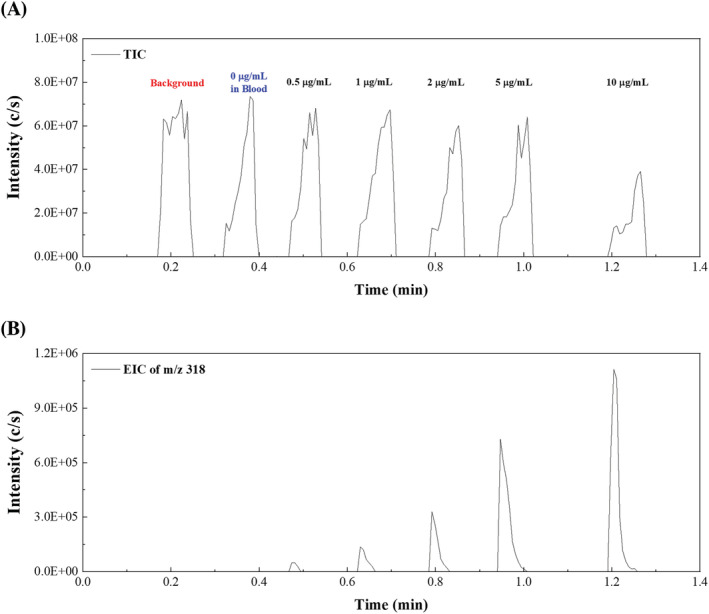
Sheath‐flow probe electrospray ionisation‐mass spectrometry (sfPESI‐MS) ion chromatograms from the sequential ionisation analysis of unspiked dried blood spot and dried blood spots spiked with different concentrations (from 0.5 to 10 μg/ml) of cocaethylene (CE): A, total ion chromatogram (TIC) and B, extracted ion chromatogram (EIC) of the [CE + H]^+^ ion (*m/z* 318.17) [Color figure can be viewed at wileyonlinelibrary.com]

The rapid separation and analysis achieved using sfPESI‐MS is demonstrated in Figure 4, which shows the selected ion response for the protonated CE positive ion [CE + H]^+^ when a blank glass slide and a series of DBS spiked with different concentrations of CE ranging between 0 and 10 μg/ml were analysed. Figure 4A shows the TIC with broad, unresolved peaks showing the overlapping responses for the cocaine metabolites present in the mass spectrum and background species which originate from the dried blood matrix and the surface of the sample slide. The extracted ion trace for the [CE + H]^+^ ions shows the rapid separation of the [CE + H]^+^ ion from background species present in the sample (Figure 4B). The extracted ion trace demonstrates the potential quantitative capability of the method which was further evaluated. It is also worth noting that in this example, a background sample, blank DBS and five standards were analysed in *c*. 100 s illustrating the ability of sfPESI‐MS to conduct high throughput analysis.

### 3.4 Quantitative analysis of cocaine metabolites from DBSs

Quantitative analysis was attempted using the optimised conditions described earlier. A mixture of the three drug metabolites at a range of 0.5–10 μg/ml was spiked into the blood sample aliquots, and then 5 μl of the blood mixtures was spotted onto glass slides and allowed to dry in a dark cupboard for 48 h at 22°C ± 1 before conducting sfPESI‐MS analysis. Ten replicate analyses from spiked DBS were used for each data point, 10 replicate blank analyses were performed by analysing unspiked DBS, and outliers were removed using Dixon's Q‐test. Absolute ion intensities were used to generate calibration graphs due to the small number of points over the eluted metabolite peaks.

**FIGURE 5:**
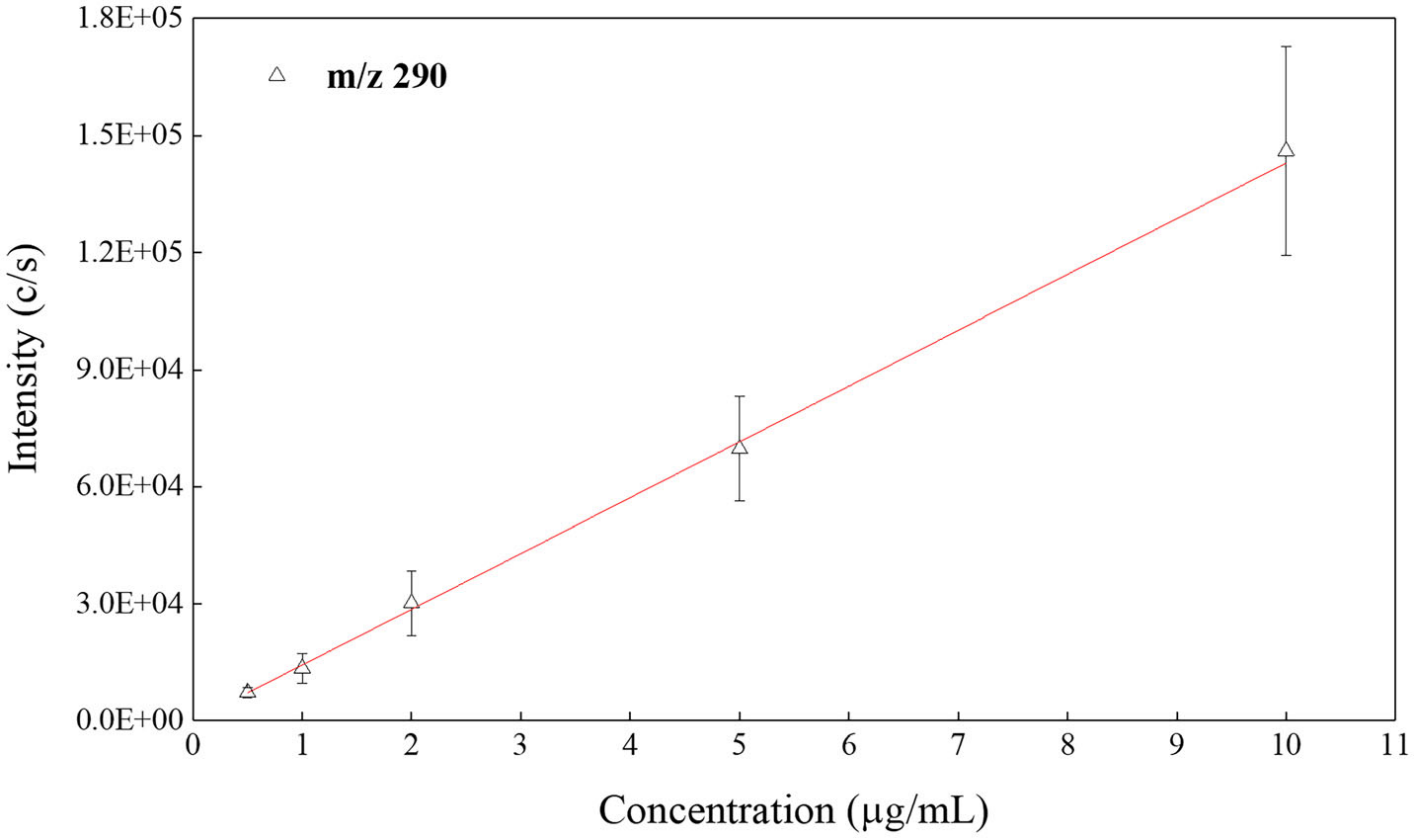
An example of calibration obtained for the [M + H]^+^ ion of benzoylecgonine (*m/z* 290.14) each data point is calculated from 10 replicate measurements (n = 10): Limit of detection (LOD) = 0.29 μg/ml and R^2^ = 0.9979 [Color figure can be viewed at wileyonlinelibrary.com]

All of the metabolites showed good quantitative performance with linear calibration graphs obtained over the range from 0.5 to 10 μg/ml for the [M + H]^+^ ions of BZE (Figure 5) and CE (Figure S2 [supporting information]). EME was not reliably detected at the 0.5 μg/ml level, and a linear correlation graph between 1 and 10 μg/ml for the [EME + H]^+^ ion was obtained (Figure S3 [supporting information]). Full details of the calibration data are shown in Table S5 (supporting information). Cocaine metabolites were not detected in any of the replicate blank measurements or in the blanks performed between sample analyses.

LODs for each of the three metabolites were calculated from the calibration data and were as follows: BZE = 0.29 μg/ml, CE = 0.15 μg/ml and EME = 1.31 μg/ml. The % RSD across the calibration range for the cocaine metabolites ranged between 19%–28% for [BZE + H]^+^, 13%–21% for [CE + H]^+^ and 12%–29% for [EME + H]^+^, and the full set of calibration data is shown in Table S5 (supporting information). These data show that the method has reasonable accuracy when analysing 48 h aged DBS for cocaine metabolites. This analysis method is fast to run 10 replicate analyses with blanks between all samples in *c*. 5 min. The main limitation of the set‐up demonstrated using the Orbitrap MS system in this manuscript is that the LODs achieved for all three metabolites were too high to detect habitual users at the blood limit for drug driving set by the UK Department for Transport (UKDfT).^36^ The UKDfT regulations classify BZE as a metabolite of an illicit drug and thus adopt a zero‐tolerance approach by setting a limit of 0.05 μg/ml in blood as determined by a gas chromatography‐mass spectrometry (GC–MS) assay, which is the highest amount of BZE in the blood that is thought to result from accidental exposure.^36^ This is below the LOD of the instrumental configuration presented here.

The instrumental set‐up as presented could potentially detect high levels of exposure and overdose in an emergency room or forensic setting, and in this potential scenario the method would be useful as it could give a rapid indication of the cause of intoxication or if a blood spot originated from an intoxicated individual. A study comparing patients who had died of a cocaine overdose and those arrested for a traffic violation while under the influence of cocaine found a median level of BZE in the blood of deceased patients of 0.7 μg/ml where cocaine was the only drug present and 0.4, 0.3 and 0.45 μg/ml, respectively, when there were 1, 2 and 3 other drugs co‐ingested with cocaine.^37^ These levels are achievable with sfPESI‐MS using the instrumental configuration described here. However, further development is required to achieve LODs that can reliably detect habitual users. Integrating the sfPESI emitter with a more sensitive MS system such as a triple quadrupole mass spectrometer (QQQ‐MS) using selected ion monitoring (SIM) in a selected reaction monitoring (SRM) experiment is recommended to address this issue.

## 4 CONCLUSIONS

As a rapid, minimally destructive analysis method, sfPESI‐MS was successfully applied to analyse three cocaine metabolites (BZE, CE and EME) directly from DBSs with no prior sample preparation. Only a small area of the DBS (≤ 1 mm^2^) was subjected to analysis leaving the rest of the sample available for analysis by other techniques including polymerase chain reaction (PCR) test and DNA analysis. The method is, in addition, fast, and a sample and blank can be analysed in less than 30 s, showing the potential of sfPESI for high throughput analysis. Adding chemical modifiers into the sfPESI probe extraction solvent was shown to enhance the stability and intensity of the mass spectral responses for all three cocaine metabolites. In standard ESI analysis adding FA and SA into the sfPESI probe solvent is shown to cause the formation of sodium formate clusters, [NaOOCH]_n_ Na^+^ (1 ≤ n), which can affect the performance of the analysis as matrix interferences. The characteristic of the sequential ionization in sfPESI‐MS analysis described by Rahman et al^22^ and Mandal et al^27^ was shown to be able to resolve cocaine metabolite ions from the [NaOOCH]_n_ Na^+^ (1 ≤ n) ions, with the metabolite ions emitted within 3 s after applying the high spray voltage (2.5 kV) to the sfPESI emitter. The rapid sequential ionization mechanism exhibited by sfPESI‐MS shows a significant benefit, promoting enhanced sensitivity by reducing in‐source ion suppression and reducing the variability of replicate measurements, which is particularly useful when using mobile phase modifiers to enable more reproducible and sensitive extraction from complex sample media such as DBS. The quantitative performance of the sfPESI‐MS method was evaluated for the three metabolites and showed adequate reproducibility (% RSD of ≤ 29%) and LODs which could be useful for the detection of overdose and/or severe intoxication in blood spots found at crime scenes. However, further development and interfacing with tandem mass spectrometry (MS/MS) instrumentation is required for the method to be able to detect users at the limit for accidental exposure.

### PEER REVIEW

The peer review history for this article is available at https://publons.com/publon/10.1002/rcm.9422.

## Supporting information


**FIGURE S1** Example of blood spots: (A) three sets of deposited dried blood spots (DBS) to analyse, (B) estimation of spatial resolution (0.03 mm^2^ ≤ area ≤ 0.79 mm^2^) of the sfPESI probe and (C) actual DBS sampling model using a sfPESI probe
**TABLE S1** Optimised parameters of Orbitrap MS
**TABLE S2** Comparative data showing the effect of extraction solvent chemical modification using 0.1% formic acid on the replicate (n = 10) sfPESI–MS analysis of a 5 μg/ml liquid standard solution containing benzoylecgonine (BZE)
**TABLE S3** Comparative data showing the effect of extraction solvent chemical modification using 0.5mM sodium acetate on the replicate (n = 10) sfPESI–MS analysis of a 5 μg/ml liquid standard solution containing benzoylecgonine (BZE)
**TABLE S4** Comparative data showing the effect of extraction solvent chemical modification using 0.5mM sodium acetate and 0.1% formic acid on the replicate (n = 10) sfPESI–MS analysis of a 5 μg/ml liquid standard solution containing benzoylecgonine (BZE), ecgonine methyl ester (EME) and cocaethylene (CE)
**FIGURE S2** An example of calibration obtained for the [M + H]^+^ ion of cocaethylene (*m/z* 318.17) each data point is calculated from 10 replicate measurements (n = 10): LOD = 0.15 μg/ml and R^2^ = 0.9948
**FIGURE S3** An example of calibration obtained for the [M + H]^+^ ion of ecgonine methyl ester (*m/z* 200.13) each data point is calculated from 10 replicate measurements (n = 10): LOD = 1.31 μg/ml and R^2^ = 0.9895
**TABLE S5** Calibration and reproducibility data obtained from replicate dried blood spot analyses (n = 10) for ecgonine methyl ester (EME), benzoylecgonine (BZE) and cocaethylene (CE)

## Data Availability

The data that support the findings of this study are available from the corresponding author upon reasonable request.
