# Supplementary material for: Development of a rapid, in‐situ analysis method using sheath‐flow probe electrospray ionisation‐mass spectrometry for the direct identification of cocaine metabolites in dried blood spots
Source: Rapid Commun Mass Spectrom. 2022 Nov 16;37(2):e9422. doi: 10.1002/rcm.9422 (PMC9788050; doi:10.1002/rcm.9422)
Supplement: Supplementary file 1 — FIGURE S1 Example of blood spots: (A) three sets of deposited dried blood spots (DBS) to analyse, (B) estimation of spatial resolution (0.03 mm2 ≤ area ≤ 0.79 mm2) of the sfPESI probe and (C) actual DBS sampling model using a sfPESI probe TABLE S1 Optimised parameters of Orbitrap MS TABLE S2 Comparative data showing the effect of extraction solvent chemical modification using 0.1% formic acid on the replicate (n = 10) sfPESI–MS analysis of a 5 μg/ml liquid standard solution containing benzoylecgonine (BZE) TABLE S3 Comparative data showing the effect of extraction solvent chemical modification using 0.5mM sodium acetate on the replicate (n = 10) sfPESI–MS analysis of a 5 μg/ml liquid standard solution containing benzoylecgonine (BZE) TABLE S4 Comparative data showing the effect of extraction solvent chemical modification using 0.5mM sodium acetate and 0.1% formic acid on the replicate (n = 10) sfPESI–MS analysis of a 5 μg/ml liquid standard solution containing benzoylecgonine (BZE), ecgonine methyl ester (EME) and cocaethylene (CE) FIGURE S2 An example of calibration obtained for the [M + H]+ ion of cocaethylene (m/z 318.17) each data point is calculated from 10 replicate measurements (n = 10): LOD = 0.15 μg/ml and R2 = 0.9948 FIGURE S3 An example of calibration obtained for the [M + H]+ ion of ecgonine methyl ester (m/z 200.13) each data point is calculated from 10 replicate measurements (n = 10): LOD = 1.31 μg/ml and R2 = 0.9895 TABLE S5 Calibration and reproducibility data obtained from replicate dried blood spot analyses (n = 10) for ecgonine methyl ester (EME), benzoylecgonine (BZE) and cocaethylene (CE) [file RCM-37-e9422-s001.docx]

**ELECTRONIC SUPPLEMENTARY MATERIAL**

**Development of a rapid *in-situ* analysis method using sheath-flow probe electrospray ionisation-mass spectrometry (sfPESI-MS) for the direct identification of cocaine metabolites in dried blood spots**

Ayoung Kim^a,^*, Paul F. Kelly^a^, Matthew A. Turner ^a^ and James C. Reynolds^a,^*.

^a^ Centre for Analytical Science, Department of Chemistry, Loughborough University, LE11 3TU, UK

* Corresponding author: [a.kim2@lboro.as.uk](mailto:a.kim2@lboro.as.uk) and [j.c.reynolds@lboro.ac.uk](mailto:j.c.reynolds@lboro.ac.uk)

1. **(b) (c)**


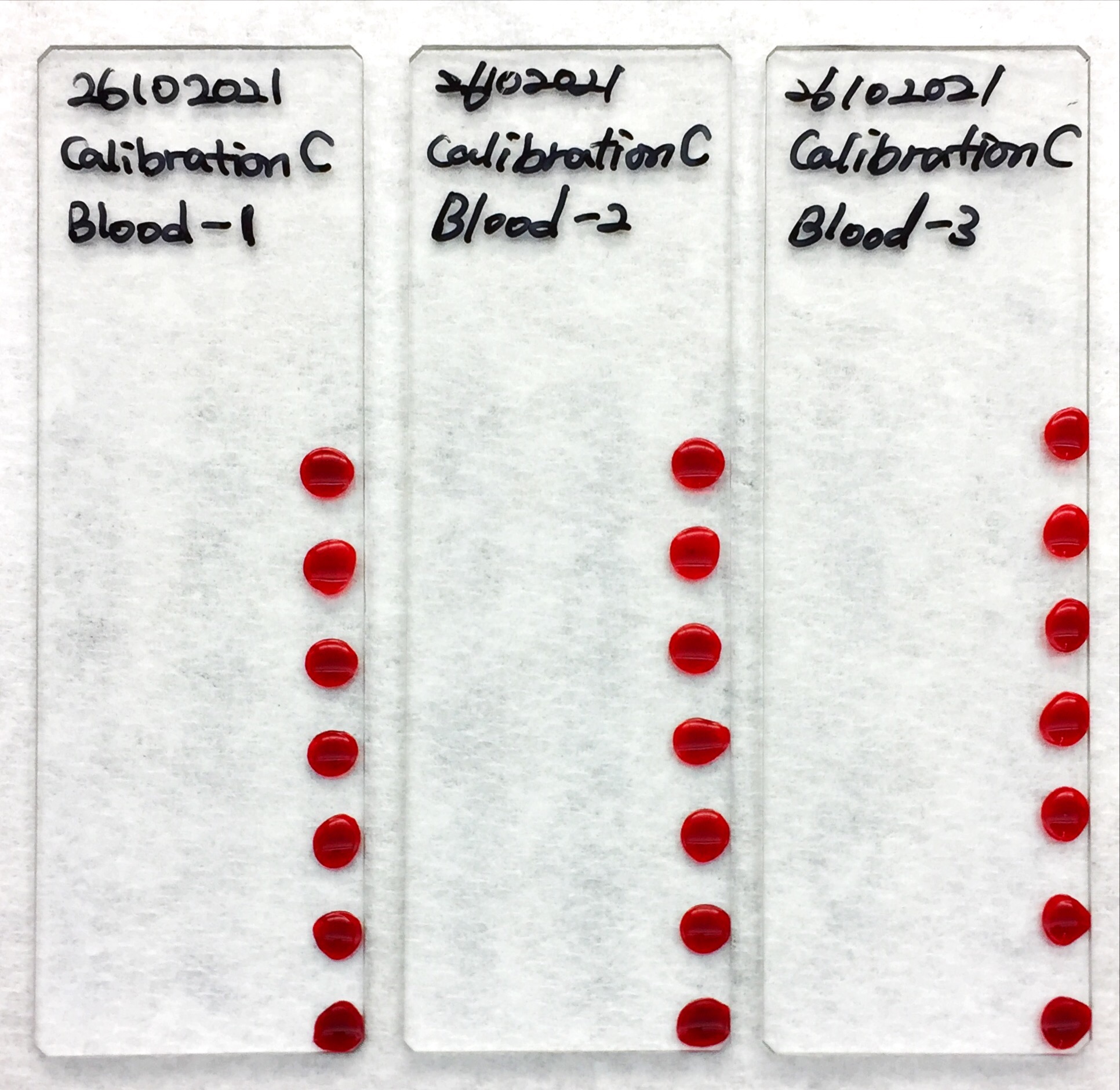

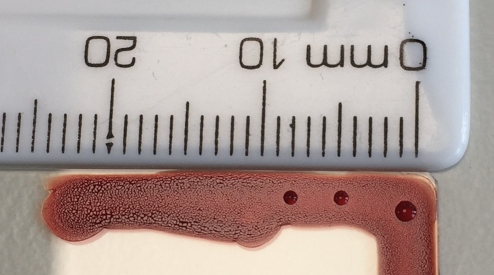

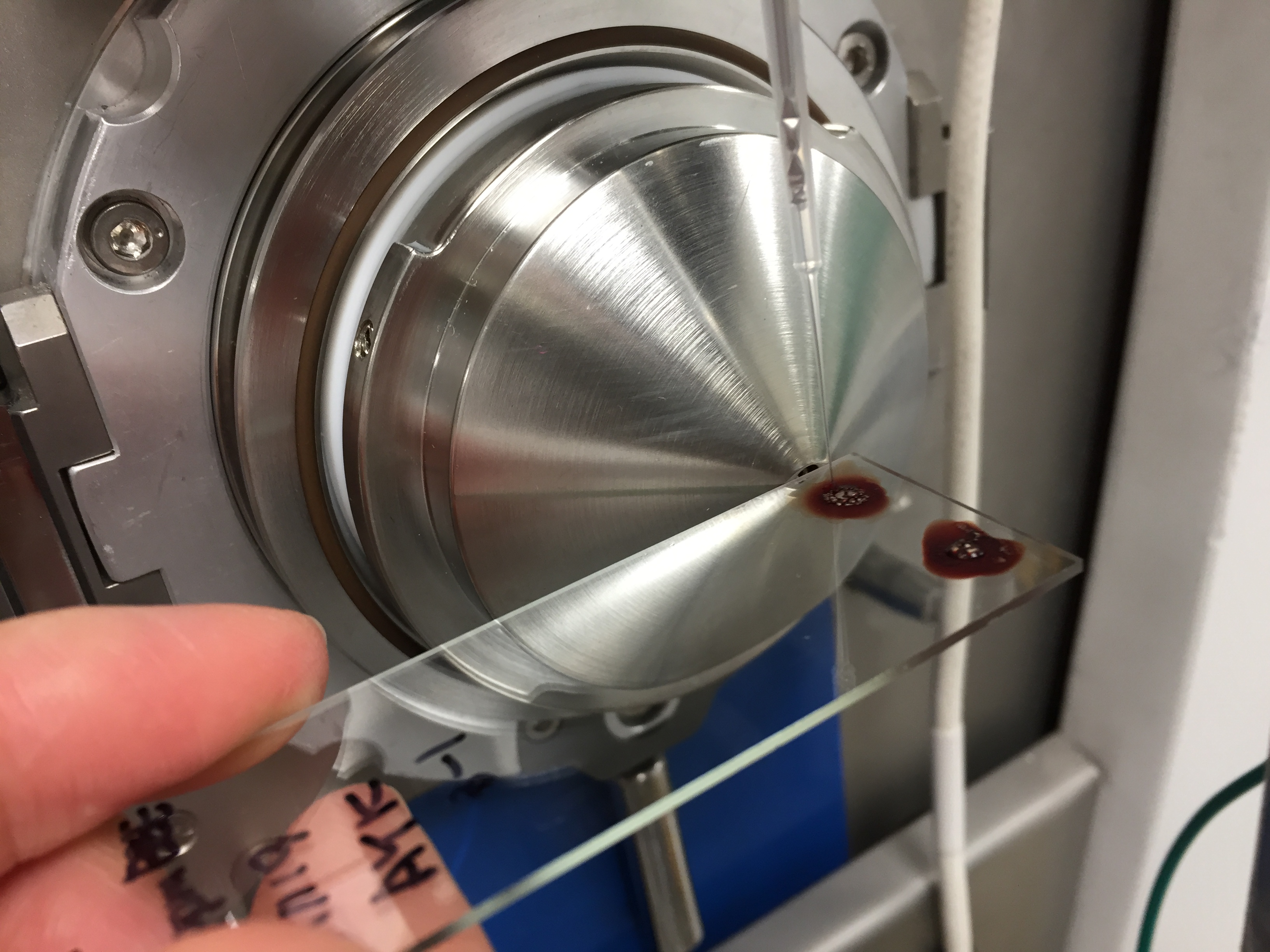


**Figure S1.** Example of blood spots: **(a)** 3 sets of deposited dried blood spots (DBS) to analyse, **(b)** estimation of spatial resolution (0.03 mm^2^ ≤ area ≤ 0.79 mm^2^) of the sfPESI probe and **(c)** actual DBS sampling model using a sfPESI probe

**Table S1.** Optimised Parameters of Orbitrap MS

| Parameters | | Values | Units |
| --- | --- | --- | --- |
| Scan  parameters | Scan Mass range | 50 - 450 | ^a^ *m/z* |
|  | Mass Resolution | 25,000 | - |
|  | Scan Rate (Average) | 2.4 | ^b^ scans/sec |
|  | Polarity | Positive | - |
| Ion source  parameters | Capillary Temperature | 300 | °C |
|  | Capillary Voltage | 32.5 | ^c^ V |
|  | Tube Lens Voltage | 90.0 | V |
|  | Skimmer Voltage | 28.0 | V |

^a^ *m/z*: mass-to-charge ratio, ^b^ sec: second and ^c^ V: volts

**Table S2.** Comparative data showing the effect of extraction solvent chemical modification using 0.1% formic acid on the replicate (n=10) sfPESI-MS analysis of a 5 μg/mL liquid standard solution containing benzoylecgonine (BZE).

| Molecular Ion Form  of adducted BZE | Target Mass | Mass Spectrum  Intensity (c/s) | Concentration of Formic Acid | | |
| --- | --- | --- | --- | --- | --- |
|  |  |  | 0 % | 0.1 % | 0.5 % |
| [M+H]^+^ | *m/z* 290 | M (n = 10) | 1.42.E+05 | 4.37.E+06 | 2.78.E+06 |
|  |  | STDEV | 1.12.E+05 | 1.88.E+06 | 1.61.E+06 |
|  |  | % RSD | 79 | 43 | 58 |
| [M+Na]^+^ | *m/z* 312 | M (n = 10) | 4.33.E+05 | 6.74.E+05 | 2.35.E+05 |
|  |  | STDEV | 3.11.E+05 | 4.32.E+05 | 1.69.E+05 |
|  |  | % RSD | 72 | 64 | 72 |
| [M+K]^+^ | *m/z* 328 | M (n = 10) | 7.79.E+04 | 1.79.E+05 | 5.84.E+04 |
|  |  | STDEV | 6.23.E+04 | 8.48.E+04 | 3.22.E+04 |
|  |  | % RSD | 80 | 47 | 55 |

M: mean or average, n: the number of measurements, STDEV: standard deviation and % RSD: relative standard deviation

**Table S3.** Comparative data showing the effect of extraction solvent chemical modification using 0.5 mM sodium acetate on the replicate (n=10) sfPESI-MS analysis of a 5 μg/mL liquid standard solution containing benzoylecgonine (BZE).

| Molecular Ion Form  of adducted BZE | | [M+H]^+^ | | [M+Na]^+^ | | [M+K]^+^ | |  |
| --- | --- | --- | --- | --- | --- | --- | --- | --- |
| Target Mass | | *m/z* 290 | | *m/z* 312 | | *m/z* 328 | | |
| Concentration of Sodium Acetate | | 0 mM | 0.5 mM | 0 mM | 0.5 mM | 0 mM | 0.5 mM | |
| Mass  Spectrum  Intensity  (c/s) | M (n = 10) | 1.42.E+05 | 1.38.E+04 | 4.33.E+05 | 1.20.E+06 | 7.79.E+04 | 4.45.E+04 | |
|  | STDEV | 1.12.E+05 | 3.31.E+03 | 3.11.E+05 | 3.84.E+05 | 6.23.E+04 | 1.69.E+04 | |
|  | % RSD | 79 | 24 | 72 | 32 | 80 | 38 | |

M: mean or average, n: the number of measurements, STDEV: standard deviation and % RSD: relative standard deviation

mM(10^-3^ mol/L): millimolar (Molar concentration is units of moles of solute per litre of solution)

**Table S4.** Comparative data showing the effect of extraction solvent chemical modification using 0.5 mM sodium acetate and 0.1% formic acid on the replicate (n=10) sfPESI-MS analysis of a 5 μg/mL liquid standard solution containing benzoylecgonine (BZE), ecgonine methyl ester (EME) and cocaethylene (CE).

| Molecular Ion Form | | [BZE+H]^+^ | | [BZE+Na]^+^ | | [EME+H]^+^ | | [CE+H]^+^ | |  |
| --- | --- | --- | --- | --- | --- | --- | --- | --- | --- | --- |
| Target Mass | | *m/z* 290 | | *m/z* 312 | | *m/z* 200 | | *m/z* 318 | | |
| Chemical Modifier | | None | 0.1% FA  0.5mM SA | None | 0.1% FA  0.5mM SA | None | 0.1% FA  0.5mM SA | None | 0.1% FA  0.5mM SA | |
| Mass  Spectrum  Intensity  (c/s) | M (n=10) | 6.21E+05 | 2.07E+06 | 1.23E+05 | 4.09E+05 | 1.57E+06 | 3.54E+06 | 8.54E+05 | 1.88E+06 | |
|  | STDEV | 5.04E+05 | 6.66E+05 | 8.03E+04 | 1.58E+05 | 8.65E+05 | 1.07E+06 | 8.42E+05 | 6.84E+05 | |
|  | % RSD | 81 | 32 | 65 | 39 | 55 | 30 | 99 | 36 | |

M: mean or average, n: the number of measurements, STDEV: standard deviation and % RSD: relative standard deviation

mM(10^-3^ mol/L): millimolar (Molar concentration is units of moles of solute per litre of solution)

**Figure S2.** An Example of calibration obtained for the [M+H]^+^ ion of cocaethylene (*m/z* 318.17) each data point is calculated from 10 replicate measurements (n=10): LoD = 0.15 μg/mL and R^2^ = 0.9948.

**Figure S3.** An Example of calibration obtained for the [M+H]^+^ ion of ecgonine methyl ester (*m/z* 200.13) each data point is calculated from 10 replicate measurements (n=10): LoD = 1.31 μg/mL and R^2^ = 0.9895.

*The circled point at 1 μg/mL was determined to be below the experimental limit-of-detection as determined by 3σ/m (σ: the standard deviation in the sample response at the lowest sample concentration and m: the slope value of the linear correlation graph).

**Table S5.** Calibration and reproducibility data obtained from replicate dried blood spot analyses (n=10) for ecgonine methyl ester (EME), benzoylecgonine (BZE) and cocaethylene (CE)

| Metabolite | Concentration | 0 µg/mL | 0.5 µg/mL | 1 µg/mL | 2 µg/mL | 5 µg/mL | 10 µg/mL |
| --- | --- | --- | --- | --- | --- | --- | --- |
| EME  *m/z* 200.13 | M (n=10) | 0 | 7.33E+03 | 1.26E+04 | 1.55E+04 | 4.46E+04 | 7.05E+04 |
|  | STDEV | 0 | 0.00E+00 | 3.36E+03 | 1.91E+03 | 1.31E+04 | 1.05E+04 |
|  | % RSD | 0 | 0 | 27 | 12 | 29 | 15 |
| BZE  *m/z* 290.14 | M (n=10) | 0 | 7.24E+03 | 1.35E+04 | 3.02E+04 | 6.97E+04 | 1.46E+05 |
|  | STDEV | 0 | 1.36E+03 | 3.81E+03 | 8.40E+03 | 1.34E+04 | 2.67E+04 |
|  | % RSD | 0 | 19 | 28 | 28 | 19 | 18 |
| CE  *m/z* 318.17 | M (n=10) | 0 | 4.03E+04 | 1.00E+05 | 2.05E+05 | 5.50E+05 | 9.78E+05 |
|  | STDEV | 0 | 5.31E+03 | 1.56E+04 | 3.35E+04 | 1.17E+05 | 1.83E+05 |
|  | % RSD | 0 | 13 | 16 | 16 | 21 | 19 |

M: mean or average, n: the number of measurements, STDEV: standard deviation and % RSD: relative standard deviation
